# Advanced Liquid-Entrapped Nanosurfaces for Optimized Atmospheric Water Harvesting

**DOI:** 10.1021/acs.langmuir.4c03851

**Published:** 2024-12-20

**Authors:** Ghulam Mohd, Saswati Priyadarshini, Abhigith Nair, Versha Chauhan, Irfan Majeed Bhat, Ahmad Illahie Tantry, Shafeer Kalathil, Kowsar Majid, Saifullah Lone

**Affiliations:** † Department of Chemistry, National Institute of Technology (NIT), J&K, Srinagar, India, 190006; ‡ iDREAM (Interdisciplinary Division for Renewable Energy & Advanced Materials) NIT, J&K, Srinagar, India, 190006; § Smart Materials and Surfaces Laboratory, Faculty of Engineering and Environment, Northumbria University, Newcastle upon Tyne, NE1 8ST, U.K.; ∥ Faculty of Health and Life Sciences, Department of Applied Sciences, Northumbria University, Newcastle upon Tyne, NE1 8ST, U.K.

## Abstract

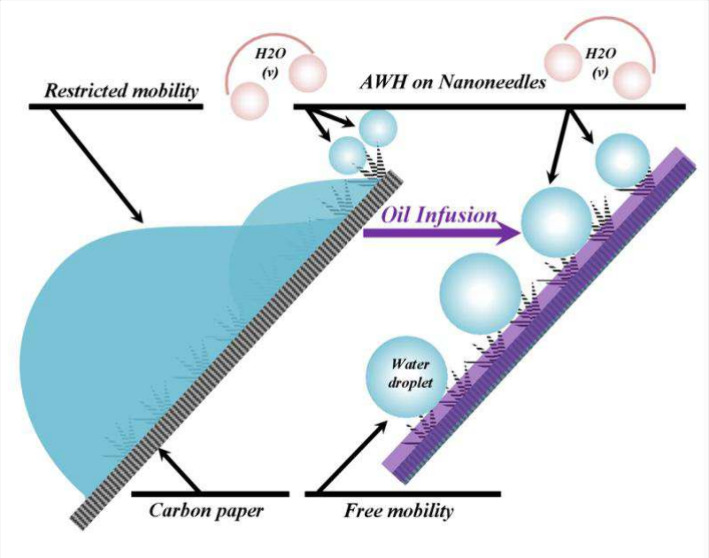

Our study addresses the pressing global freshwater scarcity crisis by engineering advanced liquid-entrapped nanosurfaces optimized for highly efficient atmospheric water harvesting (AWH). Through a synergistic approach integrating carbon fiber paper (CFP), hydrothermally synthesized nanoneedles (NNs), and silicone oil liquid entrapment (LE) within NNs, we achieved remarkable improvements in water collection efficiency. While CFP captures fog effectively during AWH, it faces challenges with water-pinning effects, mitigated by NNs’ improved droplet-spreading properties, leading to a notable 50% increase in harvesting efficiency. Further enhancements are observed upon silicone oil entrapment within CFP-bearing NNs, resulting in exceptional performance compared to noninfused surfaces. The resultant liquid entrapped nanoneedles (LE-NNs) and liquid entrapped oxidized (LE-ONNs) surfaces exhibit significant fog harvesting capability, achieving an impressive water collection rate of 21.643 ± 0.538 L/m^2^/h, which represents a 4-fold increase compared to CFP alone. This experiment was conducted with a sample area of 0.5 cm^2^. The samples were tilted at different angles to optimize mist contact with the surface, and the humidifier nozzle was positioned approximately 5 cm from the test surface to ensure a minimal fog velocity. Comprehensive analysis of morphological and compositional attributes is conducted by using techniques such as field emission scanning electron microscopy (FESEM), X-ray photoelectron spectroscopy (XPS), energy dispersive X-ray spectroscopy (EDS), and Fourier transform infrared (FTIR) spectroscopy. Leveraging CFP, NNs, or ONNs with LE presents a straightforward and highly effective surface engineering method. This approach holds promise for advancing water collection technologies and addressing global water crises sustainably.

## Introduction

Access to clean and potable water is a critical concern in many regions worldwide, particularly in arid and semiarid areas.^1,2^ A significant portion of the global population lives in water-scarce regions, facing limited access to safe drinking water.^3^ This crisis transcends geographical boundaries, impacting both developing and developed nations.^4^ Factors such as rapid population growth, poor resource management,^5^ and pollution exacerbate the situation, while climate change further complicates water scarcity through altered precipitation patterns and increased drought frequency.^6−8^ Human activities, including industrial processes and agricultural runoff, contribute to water source contamination, leading to severe health risks and reduced availability of clean drinking water.^9,10^

Certain species in nature have evolved specialized surface structures to harvest water from fog in arid environments, typically featuring microscopic bumps or grooves at the nanoscale alongside larger micro- or millimeter-scale features. For instance, cacti possess grooved, waxy spines that guide dew toward their roots for absorption.^11^ Desert moss, *Syntrichia caninervis*, is covered in nanoscale hair-like structures that capture moisture from dew and fog, delivering it to the plant’s base.^12^ Similarly, in our previous publication, Trifolium leaves have microhairs with longitudinal nanogrooves that promote droplet coalescence and movement.^13^ The Namib Desert beetle exhibits hydrophilic bumps on its back, which attract water droplets from fog; these droplets then slide down hydrophobic pathways for collection.^14,15^ These adaptations rely on gradients in surface-free energy and Laplace pressure to optimize water harvesting.^16^

Inspired by these natural mechanisms, researchers are developing synthetic surfaces designed to enhance fog collection efficiency,^17,18^ targeting areas with limited water access.^19,20^ The aim is to replicate the intricate structures found in nature using materials such as polymers and engineered metal oxides, which incorporate microscale and nanoscale features essential for condensing moisture and promoting droplet growth.^21−23^ These bioinspired surfaces offer significant potential for sustainable water harvesting in water-stressed regions.^24−26^

Despite advancements in synthetic fog-collecting surfaces, challenges remain in adapting materials and structures to various fog conditions, scaling technology for practical use, and ensuring cost-effectiveness and durability.^27^ Effective directional transport of droplets is crucial to optimize water collection and mitigate blockage at nucleation sites.^28,29^ Factors like air humidity, temperature, and geographic location significantly influence the efficacy of atmospheric water harvesting systems.^30^ Ongoing research is essential to refine design, materials, and operational parameters to ensure reliable performance in real-world scenarios.^31^ The continuous innovation in this field is poised to lead to more efficient fog-harvesting systems, contributing to sustainable water resource management.^32^

This study presents an innovative approach to fabricating a highly efficient surface for atmospheric water harvesting, aimed at addressing critical water scarcity challenges in arid and semiarid regions. The core innovation lies in the application of Ni/Co-(OH)_2_CO_3_ and NiCo_2_O_4_ nanoneedles grown on carbon fiber paper, leveraging an optimized and cost-effective hydrothermal process. This methodology offers substantial advantages over traditional techniques by eliminating additional synthesis or oxidation steps, thus streamlining production while enhancing the surface’s hydrophilic properties. Integrating silicon oil infusion further amplifies fog collection efficiency, as the infused surface supports rapid droplet coalescence and transport, minimizing losses due to re-evaporation or inefficient drainage. This dual-functional design maximizes water capture and improves the surface’s durability and longevity, offering a robust solution for real-world applications. By enhancing fog collection efficiency and ensuring long-term operational stability, this approach effectively addresses gaps in current atmospheric water harvesting technologies, presenting a scalable and sustainable solution to global water challenges, especially in regions where conventional water sources are unreliable.

### Materials and Methods

Carbon fiber paper purchased from Global Nanotech India Pvt Ltd. was precisely cut into substrates measuring 50 × 20 mm. The specific type used was Carbon papers MGL190, which comprise carbon fibers with a diameter of 0.007 mm and lengths reaching up to 1.5 mm. These papers possess a thickness of 0.3 mm and exhibit a porosity level of 78%.

### Deposition of Ni/Co-(OH)_2_CO_3_ and NiCo_2_O_4_ nanoneedles on carbon sheet

**Figure 1:**
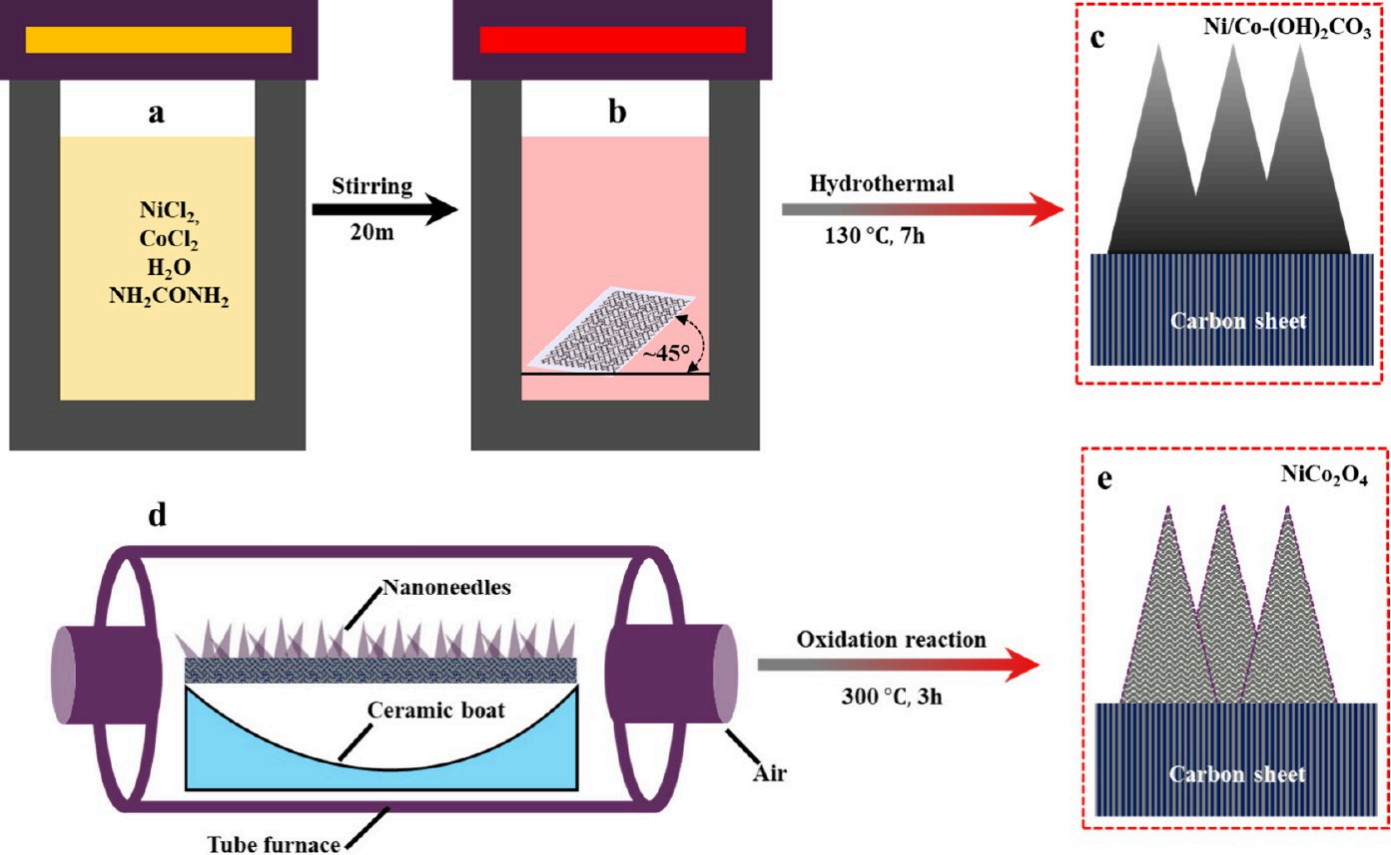
Schematic of the nanoneedle fabrication process. (a) Preparation of Ni/Co solution. (b) Immersion of carbon fiber paper in Ni/Co solution and processing in an autoclave. (c) Formation of nanoneedles (schematic diagram). (d) Thermal treatment of nanoneedles in a tube furnace. (e) Oxidized nanoneedles (schematic diagram).

In a typical procedure, 0.237 g of NiCl_2_·6H_2_O, 0.474 g of CoCl_2_·6H2O, and 0.72 g of urea were introduced into 80 mL of doubly purified deionized water in a 250 mL beaker and stirred for 20 min as shown in (Figure 1a). Afterward, we prepared a piece of acid-treated carbon sheet (2 cm × 5 cm) and firmly positioned it on a glass slide. In a standard synthesis, the nickel (Ni) and cobalt (Co) precursor compounds, consisting of 4 mmol (950.7 mg) of NiCl_2_·6H_2_O, 8 mmol (1.903 g) of CoCl_2_·6H_2_O, and 15 mmol (900.9 mg) of urea, were dissolved in 80 mL of deionized water. The resulting solution was then transferred into a 100 mL Teflon-lined autoclave, and the carbon fiber paper was positioned diagonally (45°) within the precursor solution, as shown in (Figure 1b). The setup was maintained and heated at 130 °C for 7 h in an electric oven. Following the hydrothermal treatment, the precursor, a purple suspension of Ni hydroxide carbonate, uniformly coated one side of the carbon fiber paper and was subsequently gathered. This collected material was carefully washed with deionized (DI) water and dried in a vacuum oven at 50 °C for 12 h. Subsequently, it was transferred to a tube furnace (Figure 1d) and subjected to a final calcination process at 300 °C for 3 h to transform it into black NiCo_2_O_4_ nanoneedles. The synthesis of dense, hierarchically self-assembled NiCo_2_O_4_ nanoneedles was performed using the same procedure described above.

### Surface Roughness and Wetting

**Figure 2:**
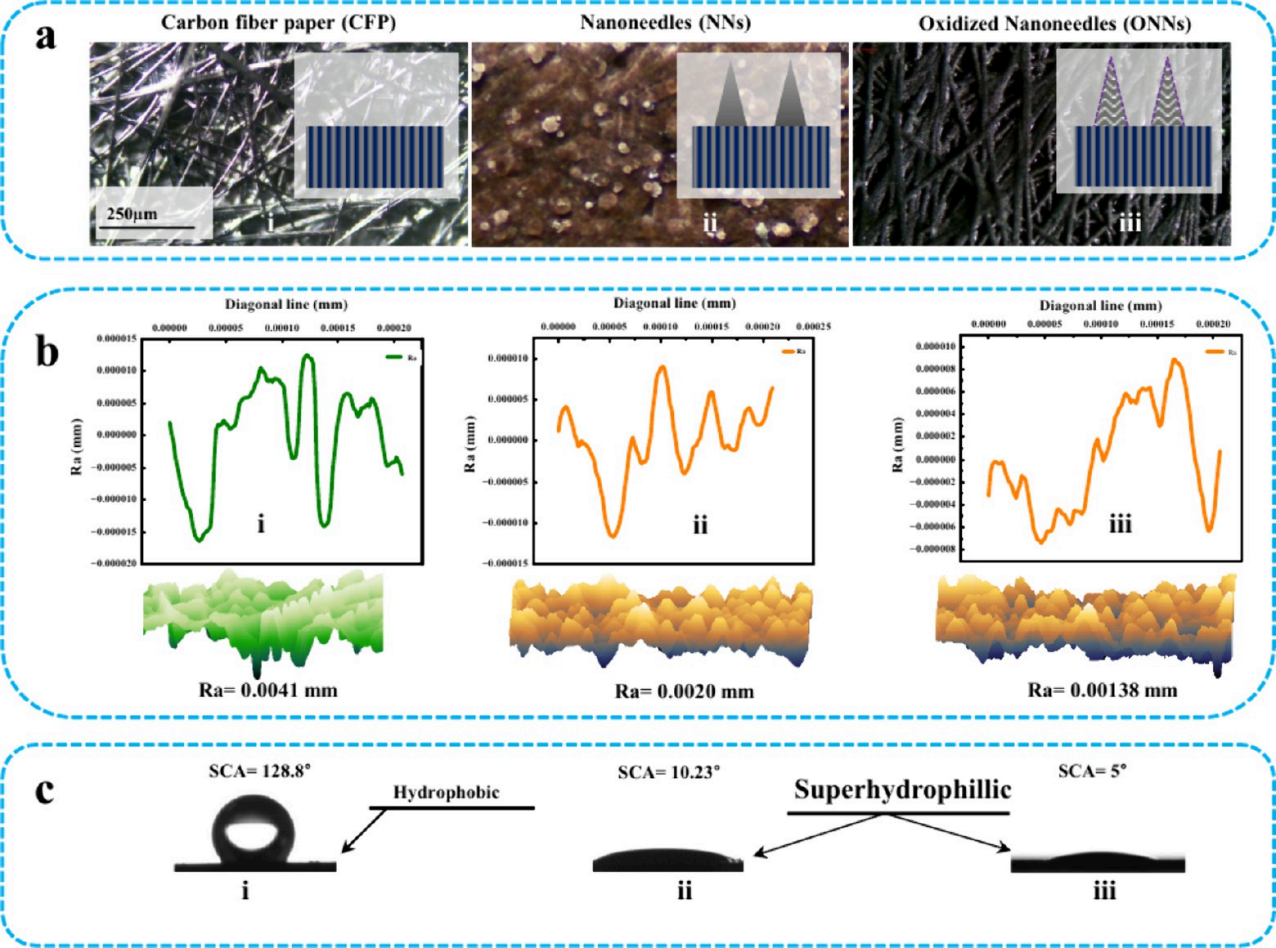
Characterization of surface roughness and wettability. (a) Microscopic visualization of surface roughness: (i) optical image of carbon fiber paper (CFP) with schematic diagram, (ii) optical image of nanoneedles (NNs), (iii) optical image of oxidized nanoneedles (ONNs). (b) 3D profilometer images and corresponding graphs: (i) 3D roughness image and graph of CFP, (ii) 3D roughness image and graph of NNs, (iii) 3D roughness image and graph of ONNs. (c) Static contact angles of (i) CFP, (ii) NNs, (iii) ONNs.

The fabrication of densely packed Ni/Co-(OH)_2_CO_3_ and NiCo_2_O_4_ nanoneedles on carbon fiber paper represents a significant advancement in surface engineering, resulting in substantial alterations in critical characteristics. This synthesis results in an increase in the surface wettability and a corresponding decrease in the contact angle, indicating more favorable interactions between the surface and liquids. Simultaneously, the insertion of nanoneedles causes subtle changes in surface roughness across multiple scales, from nano to micro. These modifications have a significant impact on the interfacial dynamics of liquids and the treated surface, influencing important parameters such as contact angle, capillary action, and total liquid–solid interaction. The wetting behavior is systematically examined using contact angle measurements, as illustrated in Figure 2. Interestingly, the presence of nanoneedles causes noticeable changes in the contact angle, transitioning the surface from hydrophobic to superhydrophilic.

### Method for Creating Hydrophobic Slippery Surfaces

The experimental procedure began by vertically positioning the samples at a precise 90° angle within a confined space of a small Petri dish. Following this, one to two drops of silicone oil (CDH, viscosity 300 cS) were meticulously applied onto the surfaces within the Petri dish. The samples were then allowed to remain in the Petri dish, facilitating the process of silicone oil infiltration into the material through capillary action, ensuring thorough permeation, as depicted inFigure 3.

**Figure 3:**
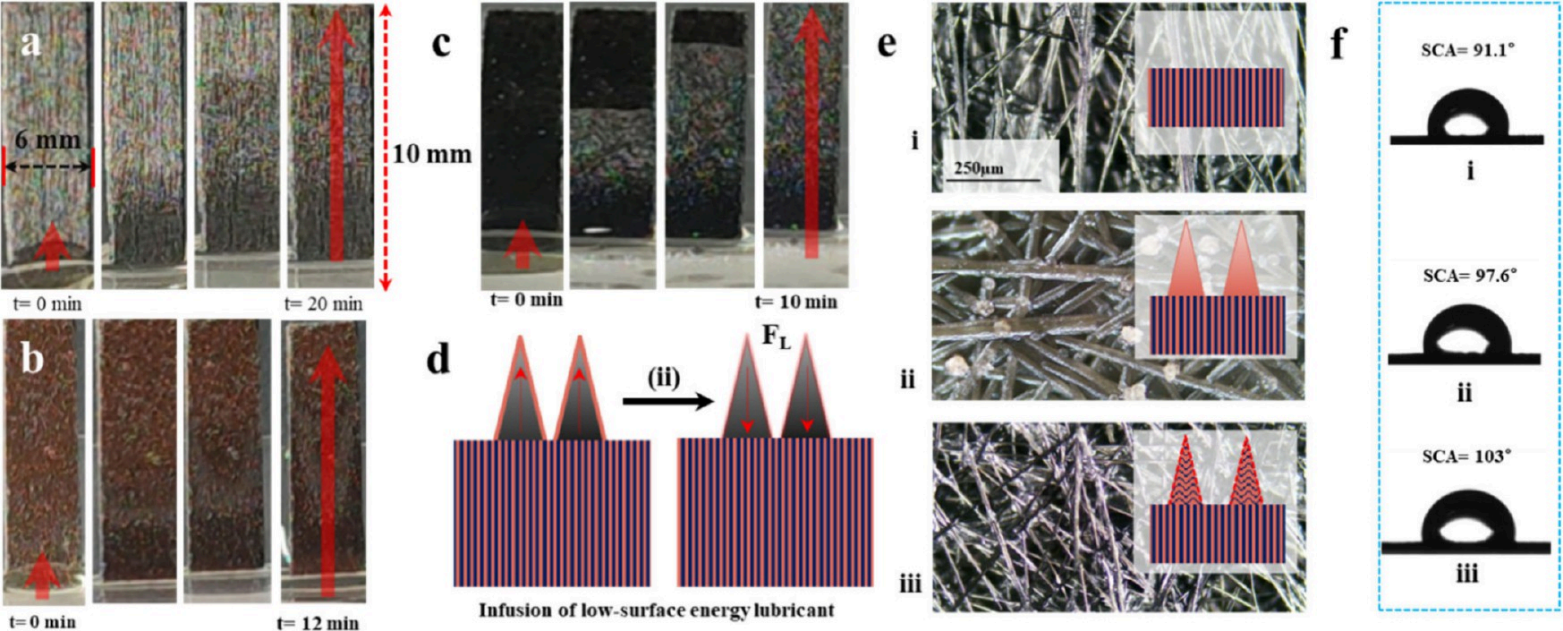
Liquid entrapped nanosurfaces. (a–c) Liquid entrapping images of the different steps of carbon fiber paper (CFP), nanoneedles (NNs), and oxidized nanoneedles (ONNs), respectively. (d) Schematic diagram of infusion. (e) Optical image of liquid entrapped nanosurface: (i) optical image of LE-CFP, (ii) optical image of LE-NNs, (iii) optical image of LE-ONNs. (f) Contact angles of liquid entrapped nanosurface: (i) contact angle image of LE-CFP, (ii) contact angle image of LE-NNs, (iii) contact angle image of LE-ONNs.

Upon achieving complete integration of the silicone oil into the samples, they were cautiously extracted from the Petri dish. Subsequently, the infused samples underwent a curing period lasting four to five hours to achieve stable oil impregnation. It is noteworthy that the samples comprised carbon fiber paper (CFP), Ni/Co-(OH)_2_CO_3_ nanoneedles (NNs) on CF, and antioxidized NiCo_2_O_4_ nanoneedles (ONNs) on CF. This meticulous procedure aimed to establish a consistent and reliable methodology for the creation of hydrophobic slippery surfaces across diverse substrate materials.

**Figure 4:**
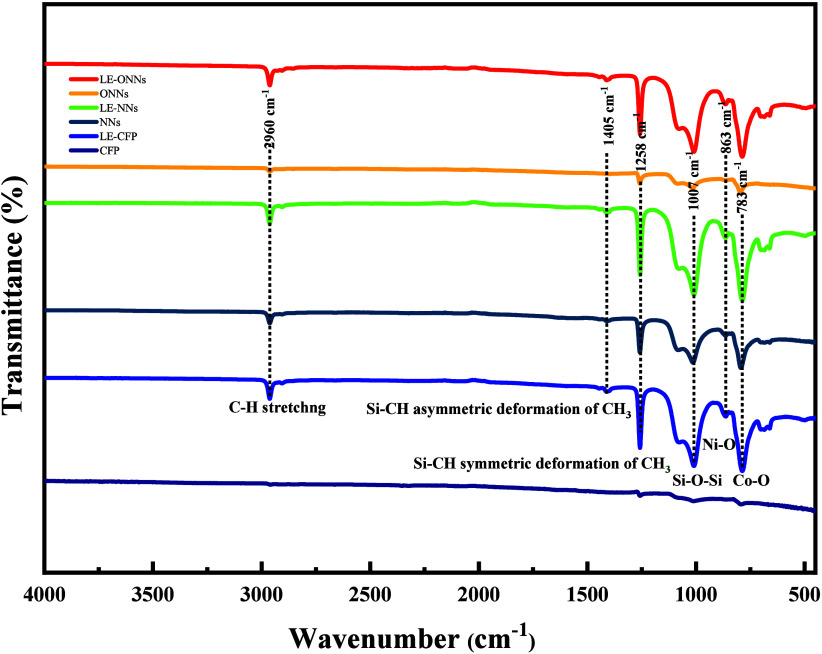
FTIR spectra of CFP (black curve), LE-CFP (green curve), NNs (blue curve), LE-NNs (purple curve), ONNs (red curve), and LE-ONNs (royal curve) papers.

Furthermore, the silicon oil coating conformation was investigated by FTIR on liquid-entrapped surfaces, as shown in Figure 4. The broad peak at 1007 cm^–1^ is representative of symmetric stretching vibrations in the Si–O–Si bonds. The silicon–oxygen–silicon network found in silicon oil coatings is responsible for this phenomenon. Additional features in the FTIR spectrum are also revealed by the analysis, most notably the presence of peaks at 1405 and 1258 cm^–1^. The peak at 1405 cm^–1^ is indicative of asymmetric deformation vibrations of Si–H bonds, providing information about the symmetry and structural configuration of the coating’s silicon, carbon, and hydrogen components. In addition, the sharp peak at 1258 cm^–1^ is correlated to the symmetric deformation vibrations of Si–CH bonds, providing further information about the symmetric features of the Si–CH moieties. These vibrational assignments highlight the molecular signatures of Si–O–Si bonds and Si–CH groups, which further contribute to a more complete understanding of the conformational characteristics of the silicon-based coating surface.

### Characterization

The analysis of contact angles at various positions on all infused and noninfused surfaces using the drop method with contact angle measuring instrument (ACAM-Series, Apex Instruments, India). The overall microstructure and interaction of water droplets with both infused and noninfused surfaces were examined using an optical microscope (LEICA, DM-600M, Germany), field emission scanning electron microscope (FESEM) was utilized to nanostructures of the nanoneedles and carbon fibers (ZEISS, GeminiSEM-500, Germany). The chemical composition underwent examination by FTIR (PerkinElmer, UATR-TWO, America), XPS (NESA G2 XPS system, America), and EDS (GeminiSEM-500, Germany).

### Fog Experiment Setup

**Figure 5:**
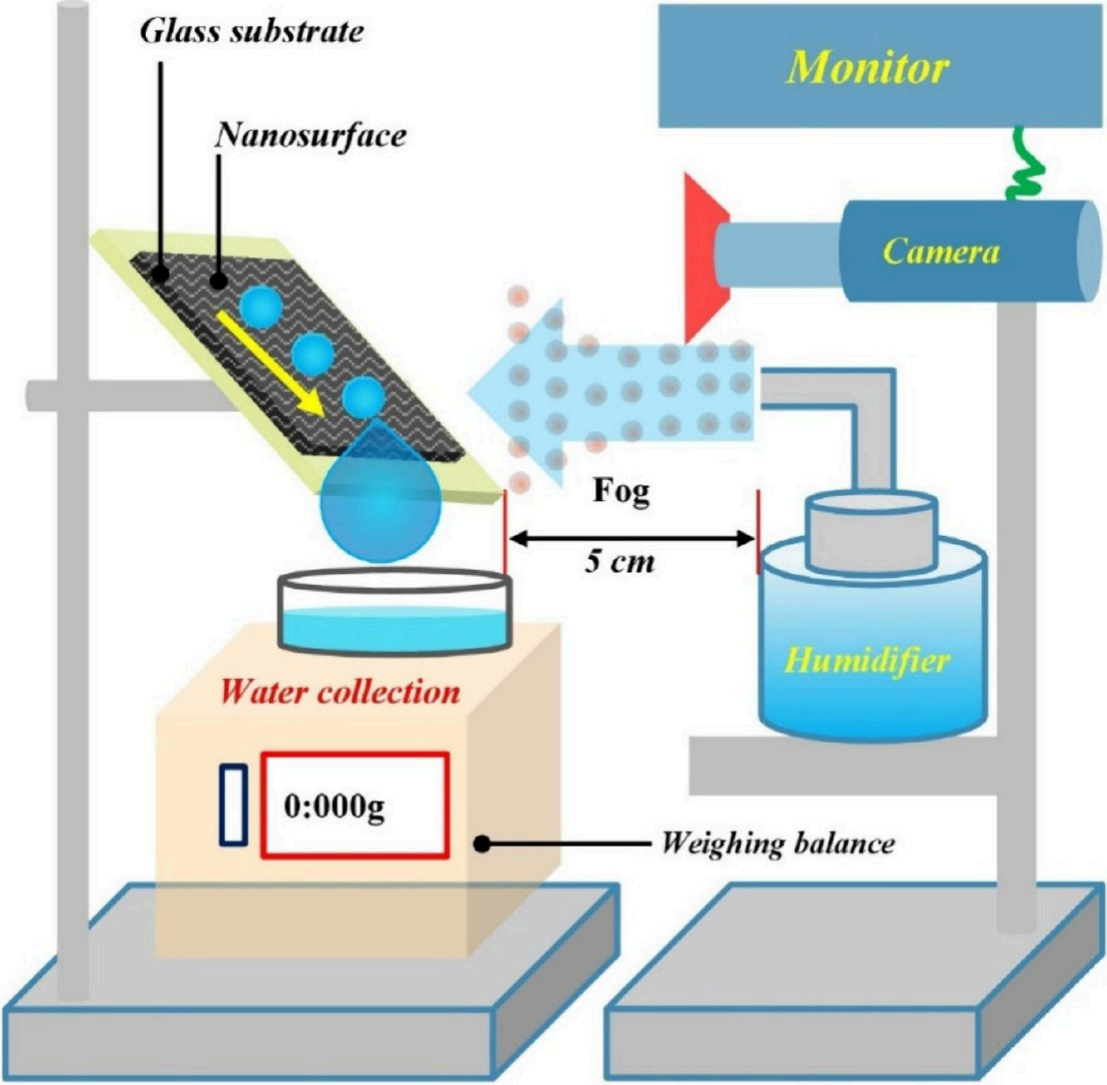
Schematic of the atmospheric water harvesting system for fog collection.

We studied how fog collects on the surfaces. For this experiment, we used a setup that involved humidifier (Allin Exporters J66 Ultrasonic Humidifier) creating a fine mist of very clean water. The samples were tilted to different angles (such as 20°, 45°, and 90°) to ensure that the mist reached the surface effectively. To ensure uniform fog distribution, the nozzle of the humidifier was positioned approximately 5 cm away from the upright test surface. We ensured that the experimental conditions remained consistent on every occasion, despite the potential for minor fluctuations in the humidifier’s output to influence the airflow. The total airflow from humidifier was about 380 mL per hour. We maintained a high humidity level (over 80%) and kept the temperature at 20 ± 5 °C throughout the experiment. The water was collected and weighed at intervals of 5, 10, 20, and 30 min using a weighing balance. In a separate aspect of the experiment, we utilized small 0.5 cm^2^ surfaces, which were freshly prepared for each trial. We performed three sets of trials using six different samples and calculated both the average values and standard deviation. Identical experimental conditions are maintained in each trial. The experimental setup is illustrated in (Figure 5).

## Results and Discussion

### Structural Characterization

**Figure 6:**
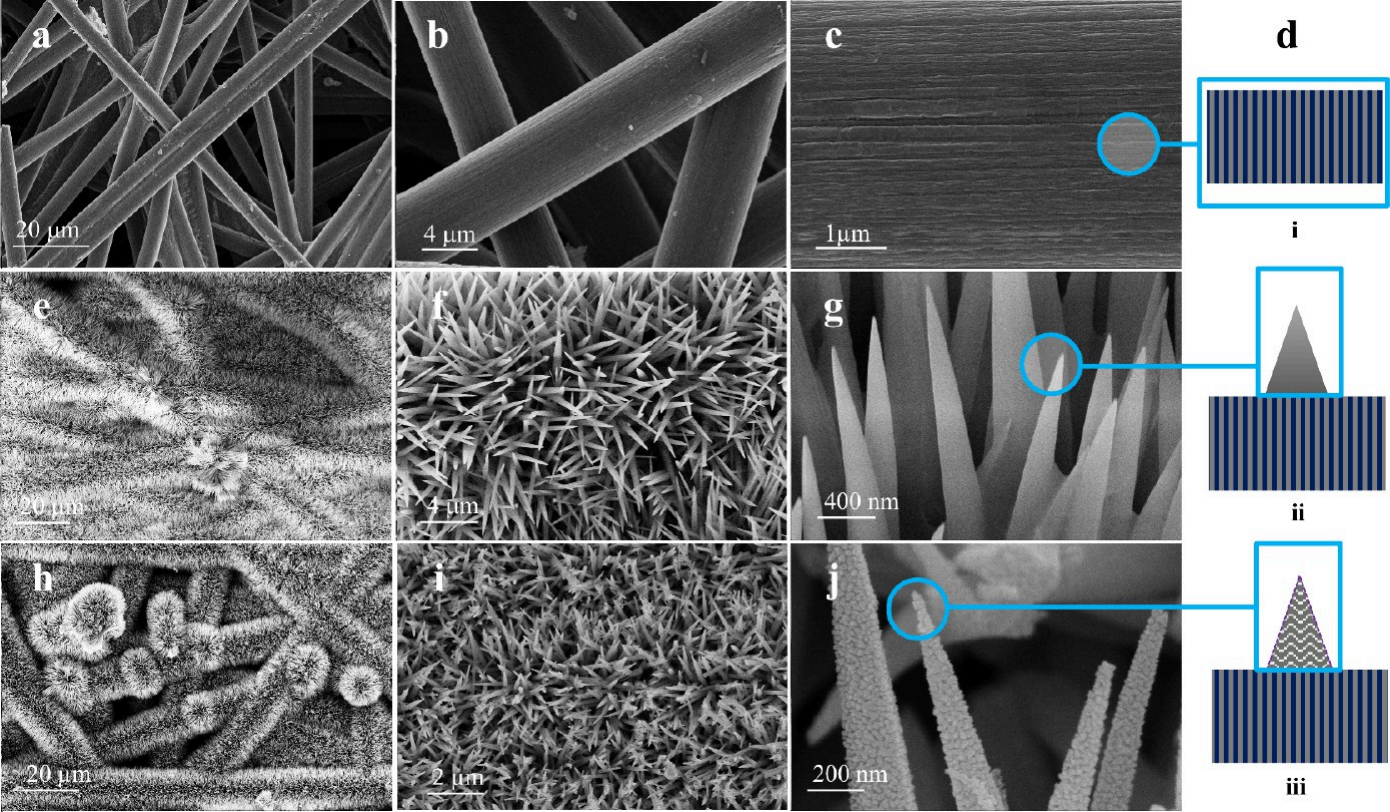
SEM topography analysis: SEM images of carbon fiber paper (CFP) (a–c), synthesized nanoneedles (NNs) (e–g), and oxidized nanoneedles (ONNs) (h–j). Panel d gives the corresponding schematic illustration of surface topography shown in panels c, g, and j.

**Figure 7:**
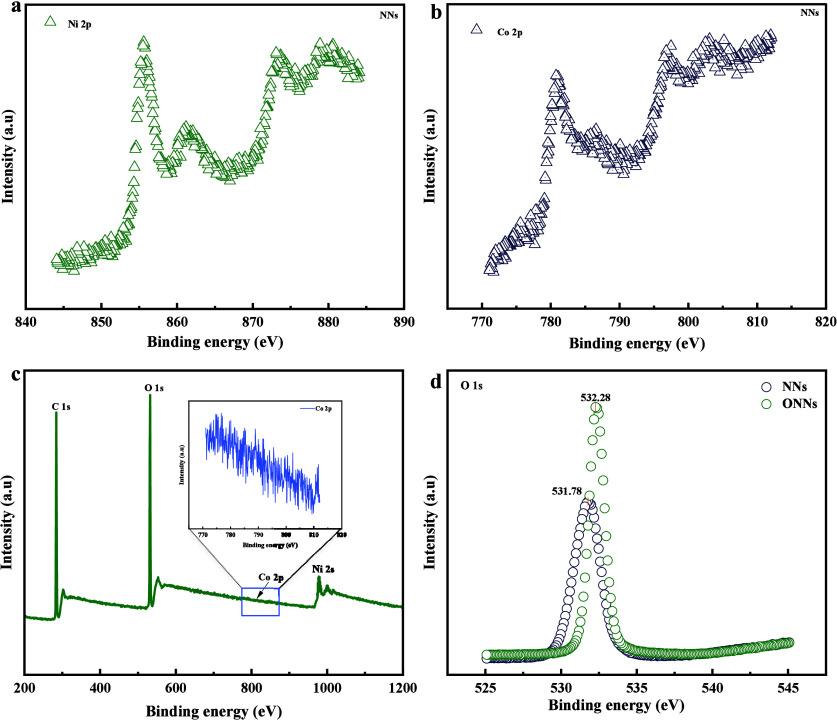
X-ray photoelectron spectroscopy (XPS) spectra: (a) Ni 2p core level XPS spectrum, (b) Co 2p core level XPS spectrum, (c) XPS spectra showing C 1s, O 1s, Co 2p, and Ni 2s core levels, and (d) the O 1s XPS spectrum.

**Figure 8:**
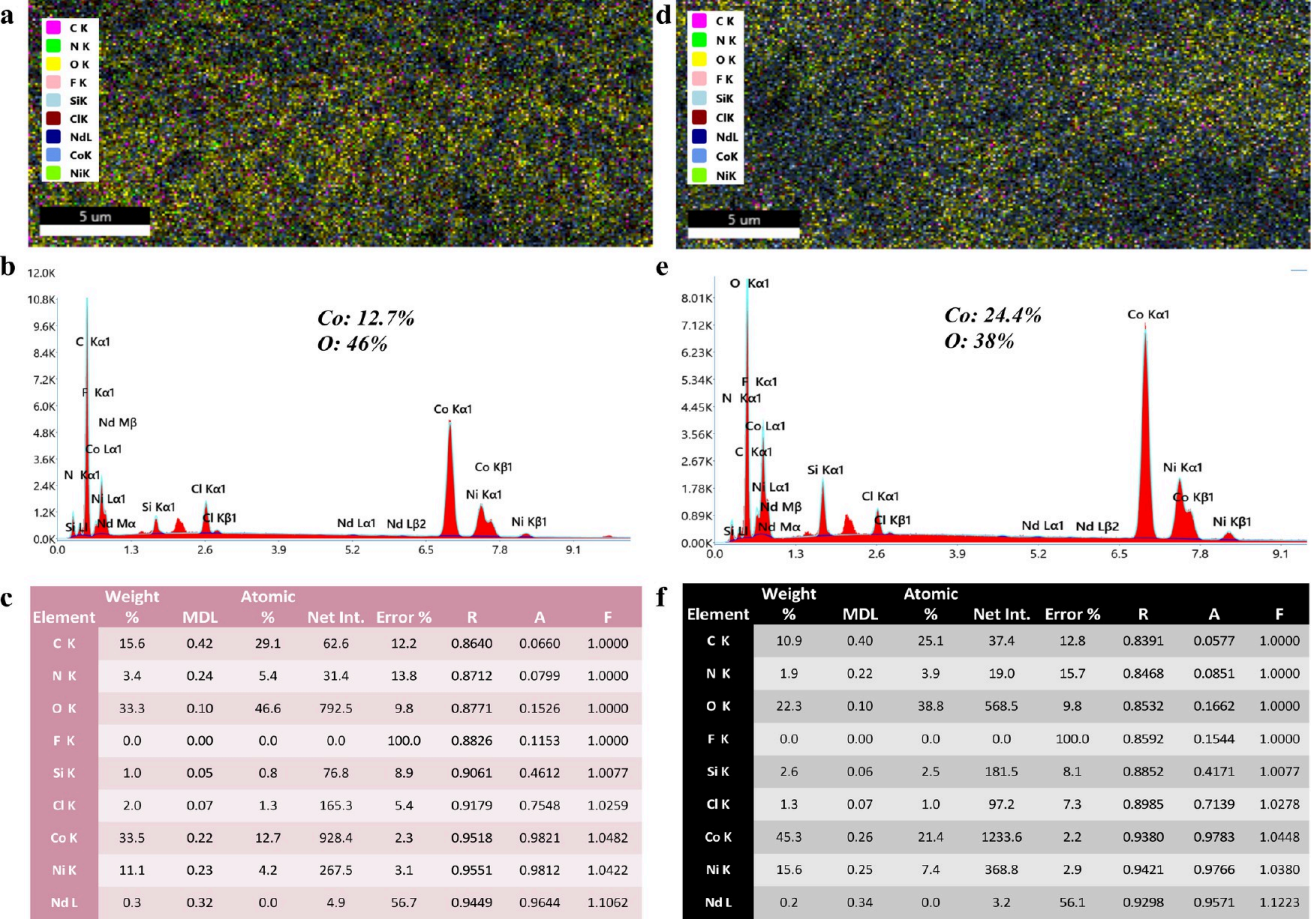
Energy dispersive X-ray (EDX) spectra analysis of NNs and ONNs surfaces. (a–c) Elemental concentration images, EDX spectra, and elemental ratios for NNs surfaces. (d–f) Elemental concentration images, EDX spectra, and elemental ratios for ONNs surfaces.

To understand the factors influencing the development of wettability modifications, we investigated the surface morphology and compositional elements, which exhibited distinct wetting behaviors. The outcomes from FESEM imaging indicate the CF, NNs and ONNs structure, nanoneedles, and density of nanoneedles are different. As delineated in (Figure 6), the nanoneedle structures of (Ni/Co–OH_2_ CO_3_) with the central region of the NNs (Figure 6e–g) display a characteristic of high density and a planar conical morphology. Conversely, in the case of the ONNs paper (Figure 6h–j), the nanoneedles (Ni/Co_2_O_4_) exhibit a comparable high density but feature a distinctively roughened conical shape. In essence, the denseness of the NNs with a rough structure corresponds to the hydrophilicity of the area. To confirm the chemical constituents, present on the surfaces X-ray photoelectron spectroscopy (XPS), energy dispersive X-ray spectroscopy (EDX), and Fourier Transform infrared (FTIR) were used. The XPS spectra for NNs surface are depicted in (Figure 7). The peaks observed at 782 and 855 eV are assigned to Co and Ni respectively, evidence points to the formation of hydrophilic (Ni/Co–OH_2_ CO_3_) nanoneedles. Observing (Figure 8), it becomes apparent that the oxygen content in NNs is 46.6%. The XPS and EDX spectra for ONNs surface are illustrated in (Figures 7 and 8). The peak observed at 812, 998, and 536 eV were assigned Co, Ni, O, and Co content exhibits a rise from 12.7% to 21.4%, indicating the formation of (Ni/Co_2_O_4_) nanoneedles. From the observation of (Figure 8), it is evident that the oxygen content in NNs is 38.8%. Additionally, to support the confirmation of the formation of both types of (Ni/Co–OH_2_Co_3_) and (Ni/Co_2_O_4_) NNs on surfaces, FTIR analysis was utilized, and the outcomes are depicted in (Figure 4). Detailed explanations of the XPS, EDX, and FTIR analyses are provided below.

(Figure 7a) X-ray photoelectron spectroscopy (XPS) spectra of Ni 2p and Co 2p core levels on the nanoneedle (NNs) surface. The Ni 2p core level XPS spectrum exhibits a peak at 855 eV, indicative of specific electronic transitions intrinsic to Ni/Co–(OH)_2_CO_3_ nanoneedles. This peak is a significant marker of the nanoneedle system’s refined electronic structure, highlighting the presence of nickel in a distinct chemical state. (Figure 7b) The Co 2p core level XPS spectrum reveals a peak at 782 eV, providing further elucidation of the structural composition of the samples. This peak corresponds to specific energy states associated with cobalt within the Ni/Co–(OH)_2_CO_3_ nanoneedles on carbon fiber paper. The observed peaks at 855 eV in the Ni 2p spectrum and 782 eV in the Co 2p spectrum uniquely identify the Ni/Co–(OH)_2_CO_3_ nanoneedle surface as the source of these signals. The detailed XPS analysis presented in (Figures 7 a and b) offers a comprehensive technical investigation of the Ni/Co–(OH)_2_CO_3_ nanoneedles on carbon fiber paper, significantly enhancing our understanding of the surface chemistry and electronic configuration of the nanoneedles through the identification of core-level peaks and their corresponding binding energies. (Figure 7c) X-ray photoelectron spectroscopy (XPS) spectra of C 1s, O 1s, Co 2p, and Ni 2s core levels provide an in-depth review of the surface chemistry of the oxidized nanoneedles (ONNs) surface. The Ni 2s and Co 2p spectra display unique characteristics indicative of the oxidation state and chemical environment of cobalt within Ni/Co_2_O_4_ nanoneedles on carbon fiber paper. The analysis of these spectra reveals distinct chemical states and bonding environments, suggesting a well-defined oxidation state of cobalt in the nanoneedles. (Figure 7d) The O 1s XPS spectra from both the NNs and ONNs surfaces reveal different oxygen species and bonding environments. This information is crucial for understanding the chemical interactions and surface composition of the nanoneedles. The detectable signals at 812, 998, and 536 eV, corresponding to Co 2p, Ni 2p, and O 1s, respectively, confirm the presence of Ni/Co_2_O_4_ nanoneedles on the carbon fiber paper. These binding energies are associated with specific electronic states of the elements, providing valuable insights into the surface chemistry and electronic structure of the nanoneedles.

The XPS analysis presented in these figures provides a critical assessment of the surface chemistry and electronic configuration of the Ni/Co-(OH)_2_CO_3_ and Ni/Co_2_O_4_ nanoneedles. The identification of core-level peaks and their corresponding binding energies elucidates the oxidation states and chemical environments of the elements, offering significant insights into the nanoneedles’ surface properties and potential applications. The study of these XPS spectra enhances our understanding of the intricate details of the nanoneedle's surface chemistry.

The energy dispersive X-ray (EDX) spectra for the nanoneedle (NNs) and oxidized nanoneedle (ONNs) surfaces reveal distinct elemental compositions, providing insights into the chemical changes resulting from the oxidation process. For the NNs surface, EDX analysis indicates a cobalt (Co) concentration of 12.7%. This concentration suggests the presence of cobalt within the initial nanoneedle structure, indicative of the Ni/Co–(OH)_2_CO_3_ phase. In contrast, the ONNs surface exhibits a higher cobalt concentration, precisely 21.4%. The increased cobalt content corresponds to the formation of Ni/Co_2_O_4_ during the oxidation process. This difference in the cobalt concentrations between NNs and ONNs points to a discernible change in the surface composition of the nanoneedles. The observed variation in cobalt concentrations is significant and highlights a clear difference in the surface composition. The transformation from Ni/Co–(OH)_2_CO_3_ in NNs to Ni/Co_2_O_4_ in ONNs is indicative of the chemical changes that occur during oxidation. This transition involves the conversion of hydroxide/carbonate species to oxides, which is reflected in the increased cobalt content observed in the ONNs.

The EDX spectra supports the hypothesis that the nanoneedle surfaces undergo distinct chemical modification during the oxidation process. The higher cobalt concentration in ONNs confirms the successful formation of a Ni/Co_2_O_4_ phase, providing the basis for the altered properties observed in oxidized nanoneedles. This detailed analysis underscores the importance of EDX in characterizing surface compositions and elucidating the chemical changes associated with nanostructure modifications as shown inFigure 8**.**

Furthermore, the confirmation was investigated by FTIR. A detailed investigation was carried out using Fourier transform infrared (FTIR) analysis, and the data obtained is graphically shown in (Figure 4). The observed spectrum features include prominent peaks at 783 and 863 cm^–1^, corresponding to vibrational modes related to metal–oxygen bonds (cobalt–oxygen, Co–O, and nickel–oxygen, Ni–O interactions, respectively). Additionally, the noticeable band at 2960 cm^–1^ is linked to the C–H stretching vibration on both surfaces. This optical property represents the molecular components present on the surfaces being studied and is identified by carbon–hydrogen bonds. The results presented above indicate that both types of NNs have differences in chemical composition. According to Wenzel’s theory, an increase in surface roughness on a hydrophilic surface corresponds to an increase in hydrophilicity. Therefore, the surface with ONNs exhibits greater hydrophilicity compared to the surface with NNs.

### Fog Harvesting Experiment

The surface topography of the CFP and surface with NNs and ONNs explored in this study exhibits two primary phases crucial for improving atmospheric water harvesting efficiency. The initial phase involves the swift nucleation, growth, and departure/shedding of water droplets on the structures. The second component is the controlled movement of water droplets to enable effective collection while maintaining the integrity of the other regions where the droplets are nucleating and growing. The water-harvesting test was carried out exactly as described in the Materials and Methods section. The distinct stages of fog harvesting experiments on various samples at various times are depicted (Figure 5).

As tiny fog droplets interact with the surface, they become visible through heterogeneous coalescence. As the fog harvesting experiment continues, droplets carried by the flowing fog combine with those already collected on the surface. In the initial stage of the experiment, all samples showed spherical droplet shapes. The droplets expand until they reach sizes similar to the capillary length (*l*_c_)^33^ equal to the given below eq 1.
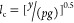
1

The surface tension between the liquid and gas phases is denoted by y; the gravitational acceleration is represented by g, and the water density is represented by p. This clearly illustrates the impact of gravitational forces. Therefore, droplet shapes are deformed, because of this gravitational pull. The hydrophilic surface shows a dominant orientation, where most droplets show a measurable orientation toward the vertical direction under the influence of gravity.

**Figure 9:**
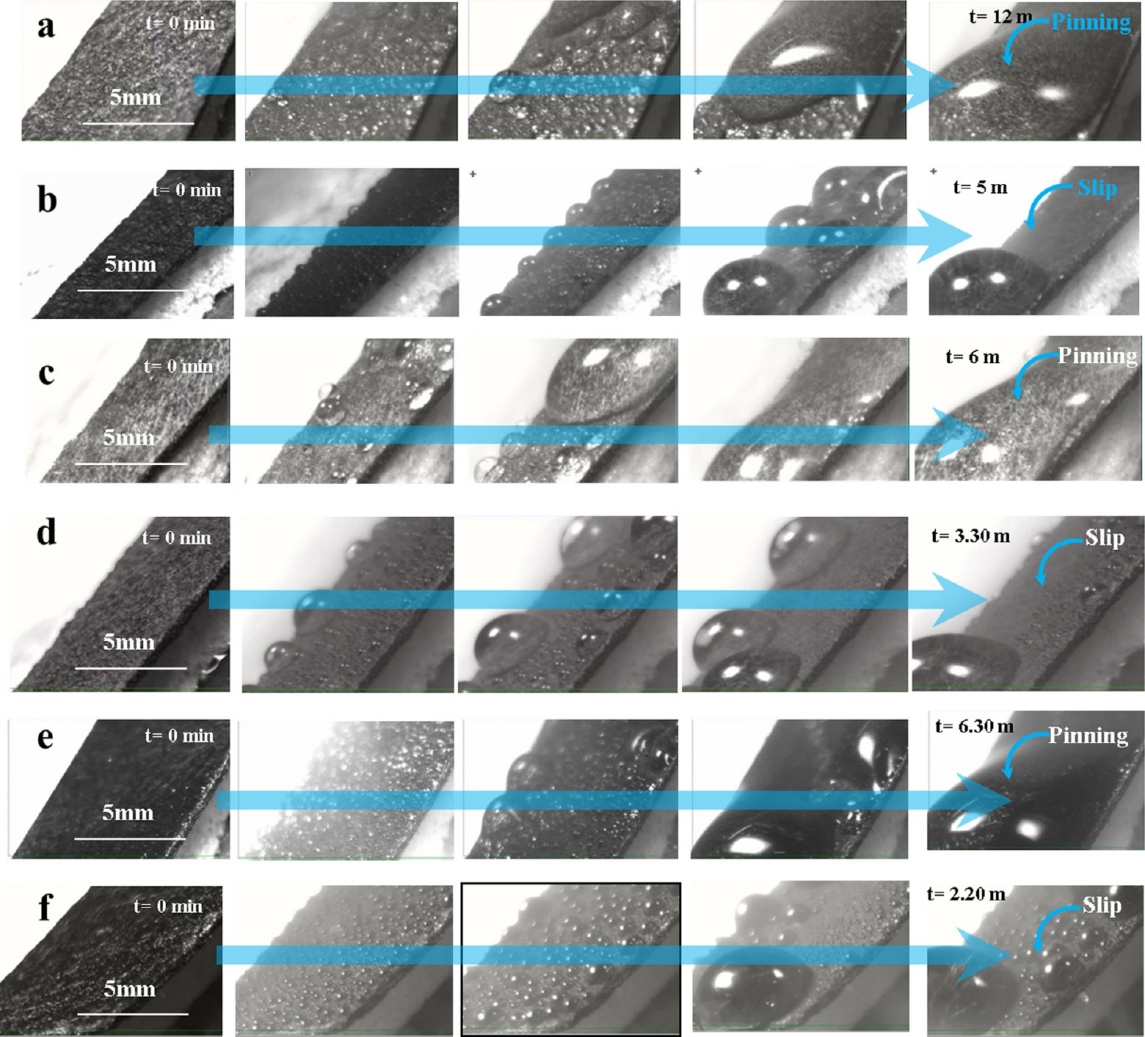
Top-view time-lapse images depicting the condensation, droplet formation, pinning and transport on various surfaces: (a) CFP, (b) LE-CFP, (c) NNs, (d) LE-NNs, (e) ONNs, (f) LE-ONNs (refer to Supporting Information S1, S2, and S3).

On the other hand, the most common behavior on carbon fiber samples without nanoneedles is the random stretching of most droplets, which leads to a film-like deposition on the surface. The unique droplet orientation observed on the hydrophilic nanoneedle surface is attributed to the geometric configuration of nanostructures in conjunction with the forces of gravity that promote droplet spreading. The various droplet morphologies observed in the current experiments necessitate larger-scale and longer-term investigations to assess the influence of vertical and horizontal forces on the droplets. Determining whether droplet spreading—which may be aided by capillary forces or other mechanisms—is related to the observed variations is still crucial. Interestingly, the liquid entrapped hydrophobic slippery surface showed constant spherical shapes throughout the experiment (Figure 9b and Supporting Information S1). However, on the liquid entrapped hydrophobic nanoneedle slippery surface, the droplets were significantly smaller and began to roll when they reached a certain size (Figure 9d and 9f, Supporting Information S2 and S3). On the other hand, in the nanoneedles (Figure 9c and 9e, Supporting Information S2 and S3) and without nanoneedles samples (Figure 9a and Supporting Information S1), the droplets experienced significant volumetric expansion and continued to adhere to the surface for a prolonged period, which could be explained by instabilities present in their Cassie states.

**Figure 10:**
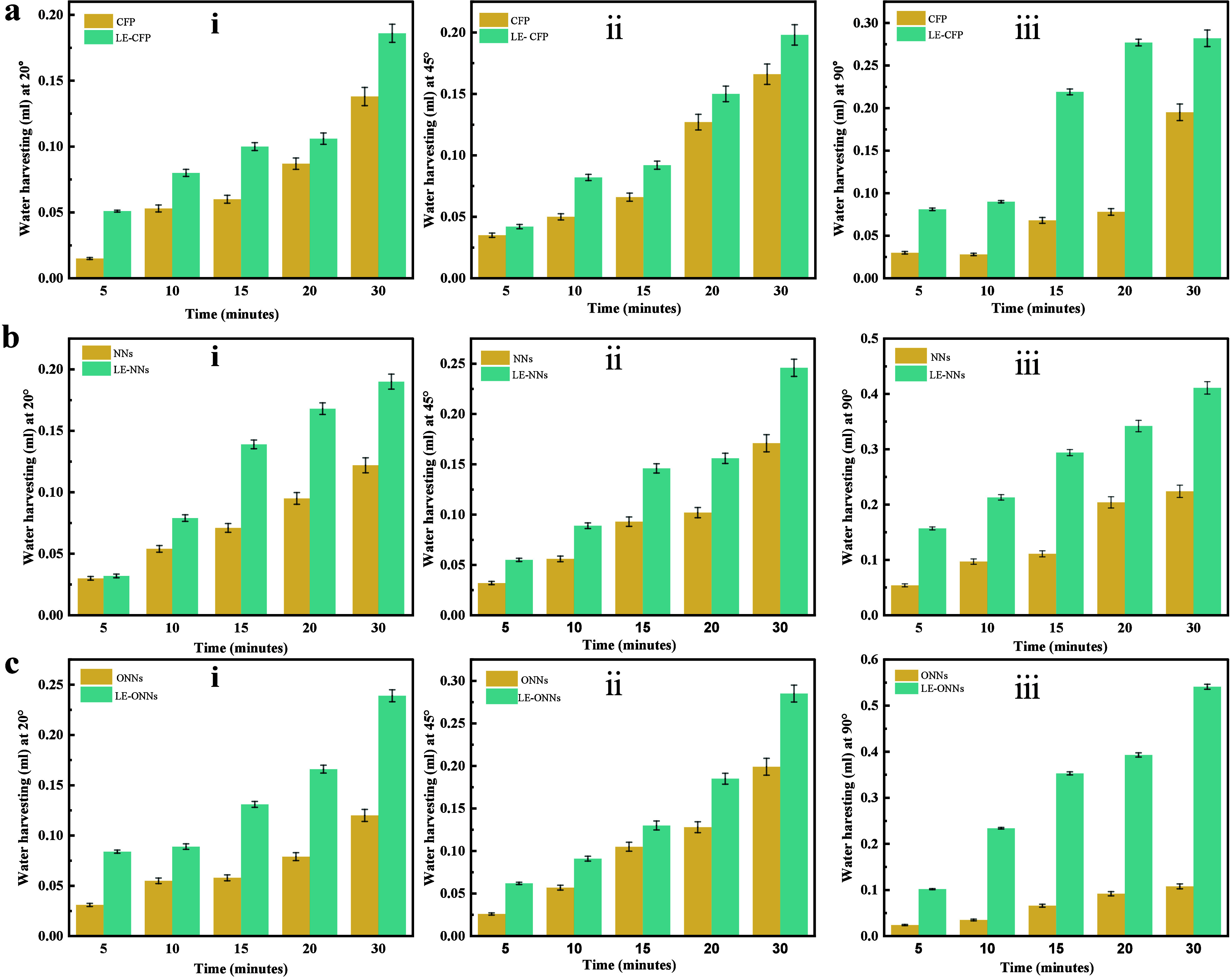
AWH performance graph of various surfaces at different angles. (a) CEF and LE-CEF: (i) at 20°, (ii) at 45°. (iii) at 90°. (b) NNs and LE-NNs: (i) at 20°, (ii) at 45°, (iii) at 90°. (c) ONNs and LE-ONNs: (i) at 20°, (ii) at 45°, (iii) at 90°.

The water volume is directly related to the effectiveness of water removal on the surfaces, because of different forces affecting droplet behavior and surface adhesion. The quantification of water dripping from the surfaces was done at 5, 10, 15, 20, and 30 min intervals to measure the water collection performance on both liquid entrapped and without-liquid entrapped surfaces at three different angles 20°, 45°, and 90° given in Figure 10a–c. Fog harvesting efficiency and surface water removal effectiveness are measured by measuring the volume and time of the falling droplets. In Figures 9 and 10, the relevant data is displayed in images and graphically. The surfaces with large droplet sizes and short onset times are thought to be better suited to fog harvesting applications. Conclusively, concerning fog collection, we consider the theoretical estimation of water available for harvesting under specific environmental conditions. We quantify the water that is accessible and can be harvested from fog. The quantification of this parameter is intricately tied to the experimental conditions employed, including factors such as humidity levels, airflow dynamics, and temperature differentials. As expected, these conditions exert a discernible influence on the efficacy of the surface water collection performance. This phenomenon serves as the predominant determinant underlying the observed disparities in water collection rate (WCR) values documented in the scientific literature. WCR, conventionally expressed as the volumetric or mass quantity of collected water per unit time per unit area, is the standard metric in these investigations.

In laboratory fog collection experiments, moisture capture typically involves using a nozzle to generate a mist spray directed onto a surface. The fog collection can fluctuate within the same setup as the mist expands (roughly conically) from the nozzle, depending on both the sample surface size and the distance between the sample and the apex of the cone. Given the circumstances, it becomes apparent that comparing results across diverse research group setups is a formidable task. Consequently, drawing comparisons between WCR values proves to be challenging. In these experiments, the surface flux distribution is nonuniform, unlike the expected homogeneity in mist velocity and fog concentration in a real natural fog collection scenario. In practical scenarios, the WCR is easily defined by dividing collected water mass per unit time by the sample area. Yet, in lab experiments, this approach is unsuitable. Thus, WCR is an insufficient metric for characterizing water collection performance, valid only under precisely replicated conditions set up. We suggest employing an efficiency ratio (η), denoting the proportion of experimentally gathered fog to the theoretically projected maximum water availability. Given in eq 2
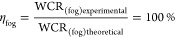
2

**Figure 11:**
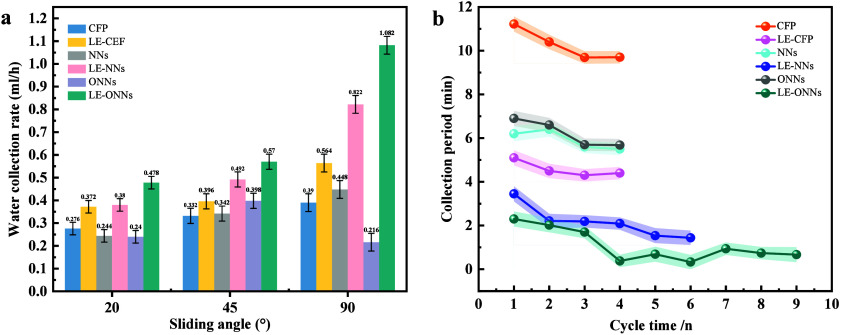
Evaluating fog collection efficacy on surfaces with diverse wettability. (a) Comparison of the fog collection efficiency of three pair surfaces such as carbon fiber paper (CFP), liquid-entrapped carbon fiber paper (LE-CFP), hydrothermally synthesized nanoneedles (NNs), oxidized nanoneedles (ONNs), and liquid entrapped nanoneedles (LE-ONNs). (b) A comparative study of cycle times on liquid entrapped nanosurface and without liquid entrapped nanosurfaces to investigate AWH dynamics.

Adhering to this methodology, the influence of varied conditions and setups enables a direct comparison of the surface performance. We have investigated the water collecting efficiency mL/hour experiment given in (Figure 11a) in the chart form.

The water collected per hour for the CFP, LE-CFP, NNs, LE-NNs, ONNs, and LE-ONNs surfaces at 20°, 45°, and 90°. To quantify the water collection rate (WCR) for these surfaces. We determine the water collection rate (WCR) by dividing the total amount of water collected over the 60 min experimental period by the product of the total sample surface area of 0.5 cm^2^ and the experimental time of 60 min at three different angles of 20°, 45°, and 90°. The CFP surface results demonstrate a WCR_(fog)_ of 5.52 L/m^2^/h at 20°, 6.64 L/m^2^/h at 45°, and 7.8 L/m^2^/h at 90°. In comparison, the LE-CFP surface exhibited a WCR_(fog)_ of 7.6 L/m^2^/h at 20°, 7.2 L/m^2^/h at 45°, and a notable increase to 11.28 L/m^2^/h at 90°. For the NNs surface, the WCR_(fog)_ values were 4.88 L/m^2^/h at 20°, 6.84 L/m^2^/h at 45°, and 8.96 L/m^2^/h at 90°. The LE-NNs surface presented WCR_(fog)_ values of 7.6 L/m^2^/h at 20°, 9.84 L/m^2^/h at 45°, and a significant rise to 16.44 L/m^2^/h at 90°. The ONNs surface had WCR_(fog)_ measurements of 4.8 L/m^2^/h at 20°, 7.96 L/m^2^/h at 45°, and 4.32 L/m^2^/h at 90°. Lastly, the LE-ONNs surface recorded WCR_(fog)_ values of 9.56 L/m^2^/h at 20°, 11.4 L/m^2^/h at 45°, and an impressive 21.64 L/m^2^/h at 90°.

Remarkably, despite being of all liquid entrapped surfaces at 20°, 45°, and 90° angles, the LE-ONNs surface at 90° angle displayed the significantly highest and best result of the overall water collection efficiency (Figure 11a). The water collection of LE-ONNs was approximately 21.643 ± 0.538 L/m^2^/h. The increased effectiveness of this surface could be attributed to its maximum active sites due to the roughness of the nanoneedles, shortened onset time, an increased rate of droplets falling, and gravity. These surfaces are also significant for the droplet’s tendency to roll off instantly, leaving the surface instantly available for further nucleation and growth cycles. This feature is visible in (Figure 11a and b).

The water harvesting efficiency cycle of surfaces without liquid entrapped is significantly limited due to surface adhesion. Water droplets stick to a surface because of the attractive forces between the water molecules and the surface material (adhesive force). This adhesion is influenced by factors such as the surface roughness, surface energy, and chemical properties. On surfaces with high surface energy or significant roughness, droplets spread more extensively and adhere more strongly, creating a larger contact area. This increased contact area leads to higher evaporation rates and reduced water collection efficiency, as shown in (Figure 11b).

In contrast, surfaces with liquid entrapped exhibit enhanced water harvesting efficiency. These surfaces are designed to be slippery with low surface energy and optimized micro- or nanostructures that reduce the contact angle of the droplets. The low surface energy minimizes water droplet adhesion, allowing droplets to form a high contact angle and exhibit minimal interaction with the surface. This reduced adhesion promotes rapid droplet rolling, which decreases the contact time between the droplet and the surface. Consequently, the water collection efficiency is improved, as droplets are collected more effectively and the system maintains higher operational efficiency, as illustrated in (Figure 11b). Thus, while without liquid-entrapped surfaces suffer from high adhesion and reduced efficiency, liquid-entrapped surfaces enhance water harvesting by minimizing droplet adhesion and improving the collection cycle.

### Wettability

The ability of a surface to retain water is contingent on its surface wettability, so we meticulously assessed the wetting characteristics of the surfaces.^34^ We investigated the wetting attributes of CFP, NNs, and LE-NNs through both droplet spreading experiments and contact angle measurements. By leveraging the knowledge of the contact angle (θ), along with the advance (*θ*_a_) and receding (*θ*_r_) contact angle, we can compute the droplet departure diameter (*D*_max_) using eq 3 where (σ) and ρ denote the surface tension and density of the liquid water, respectively, and g represents the gravitational acceleration constant.
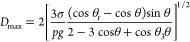
3

**Figure 12:**
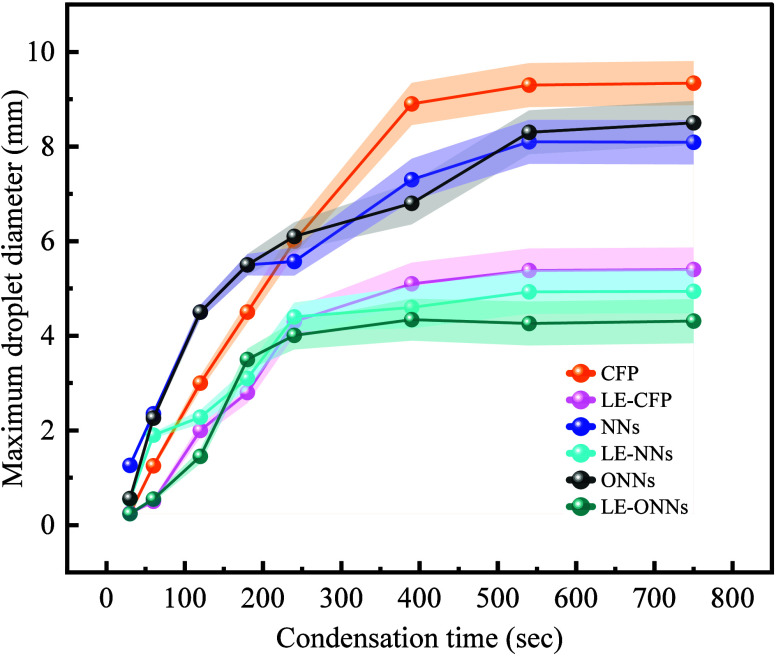
Graph depicting the temporal evolution of maximum droplet diameter, analyzed from optical microscope (OM) images by using ImageJ software.

We demonstrated that the droplet size on CFP consistently grows at least for the initial 9 min; however, the LE-ONNs surfaces show a stabilized drop diameter after two min, reaching a value within the range of 3.5 to 4 mm, emphasizing the enhanced mobility exhibited by these surfaces. Upon comparing the results elucidated in Table 1 with the graphical representation in (Figure 12), discernible conformity is evident concerning both the departing diameter and the maximal drop diameter value on the LE-ONNs surfaces. Conversely, on the non-liquid-entrapped surface, several small drops (2–4 mm in diameter) need to combine to create a drop that can slide due to gravity. Upon reaching a constant value for the maximum droplets’ diameter, this value can be considered as the departing drop diameter. This stabilization is evident on the LE-ONNs surface, occurring at about 4 mm after 750 s. Images were captured using a 10x optical microlens with the help of Capture Pro software during condensation.

**Table 1:** Diameter *D*_max_ Value of Nanosurfaces

S. No.	Type of samples	*D*_max_ (mm)
1	Carbon fiber paper (CFP)	9.3
2	Liquid entrapped carbon fiber paper (LE-CFP)	5
3	Nanoneedles (NNs)	8
4	Liquid entrapped nanoneedles (LE-NNs)	4.7
5	Oxidized nanoneedles (ONNs)	8.3
6	Liquid entrapped oxidized nanoneedles (LE-ONNs)	4

**Figure 13:**
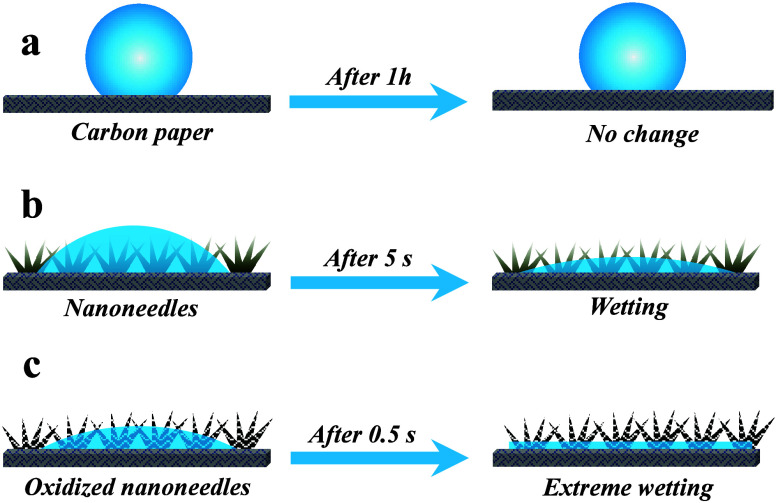
Water droplets spread as a function of time. Schematic diagram illustrating the spreading of a water droplet on (a) CFP, (b) NNs, and (c) ONNs.

It was observed that when a 5 μL water droplet was placed on the CFP surface, the water droplet did not spread out and maintained a consistent contact line of approximately the same over time, and the droplet appeared to remain spherical until evaporation (Figure 13a). We found that the CFP surface exhibited a high contact angle (>125°) and a higher contact angle hysteresis (>90°). This distinctive behavior can be attributed to the pronounced pinning effect, which is prominently evident across the entire spectrum of orientation (0° to 360°) as elucidated in (Figure 13). Further detailed information is available in (Supporting Information S4). The distinctive surface characteristics of the CFP contribute to the observed high contact angle and hysteresis, underscoring the significance of the pinning effect across diverse volumetric considerations. But after the liquid entrapped (silicon oil) both the contact angle of the surface and contact angle of hysteresis changed to 91.15° and 86.2° respectively. Details are given in Table 2.

**Table 2:**
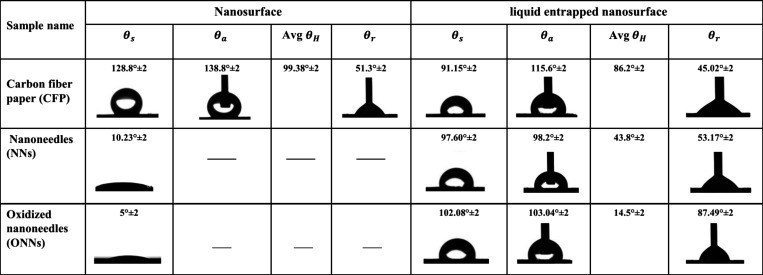
Contact Angle Analysis on Liquid Entrapped and Nanosurfaces^a^

a Including static contact angle (θ_s_), advancing contact angle (θ_a_), contact angle hysteresis (θ_H_), and receding contact angles (θ_r_). Standard deviations are provided to enhance the research precision and reliability.

In the case of the NNs and ONNs surfaces, the wetting process unfolded into two distinct phases. First phase, the droplet continued to spread slowly, completely spreading on the surface in less than 5 s (Figure 13b). In the second phase, the droplet rapidly wetted the surface and completely spread on the surface in less than 0.5 s given in (Figure 13c) for more details (see Supporting Information S5). With the immediate placement of a water droplet on the surface, a portion of the water quickly spread to the base via the tip along the nanoneedles’ surfaces. The conical nanostructures enhance the pressure gradient causing rapid wetting as soon as the droplet contacts the surface. The observed phenomenon results also the intricate interplay between capillary forces and viscous frictional forces during droplet spreading on nanostructured surfaces. In above the phase, the surface character changes from hydrophilic to hydrophobic surface after liquid entrapped silicon oil as given in Table 2. All the liquid entrapped surfaces show hydrophobic characteristics. The contact angles are greater than 90°.

**Figure 14:**
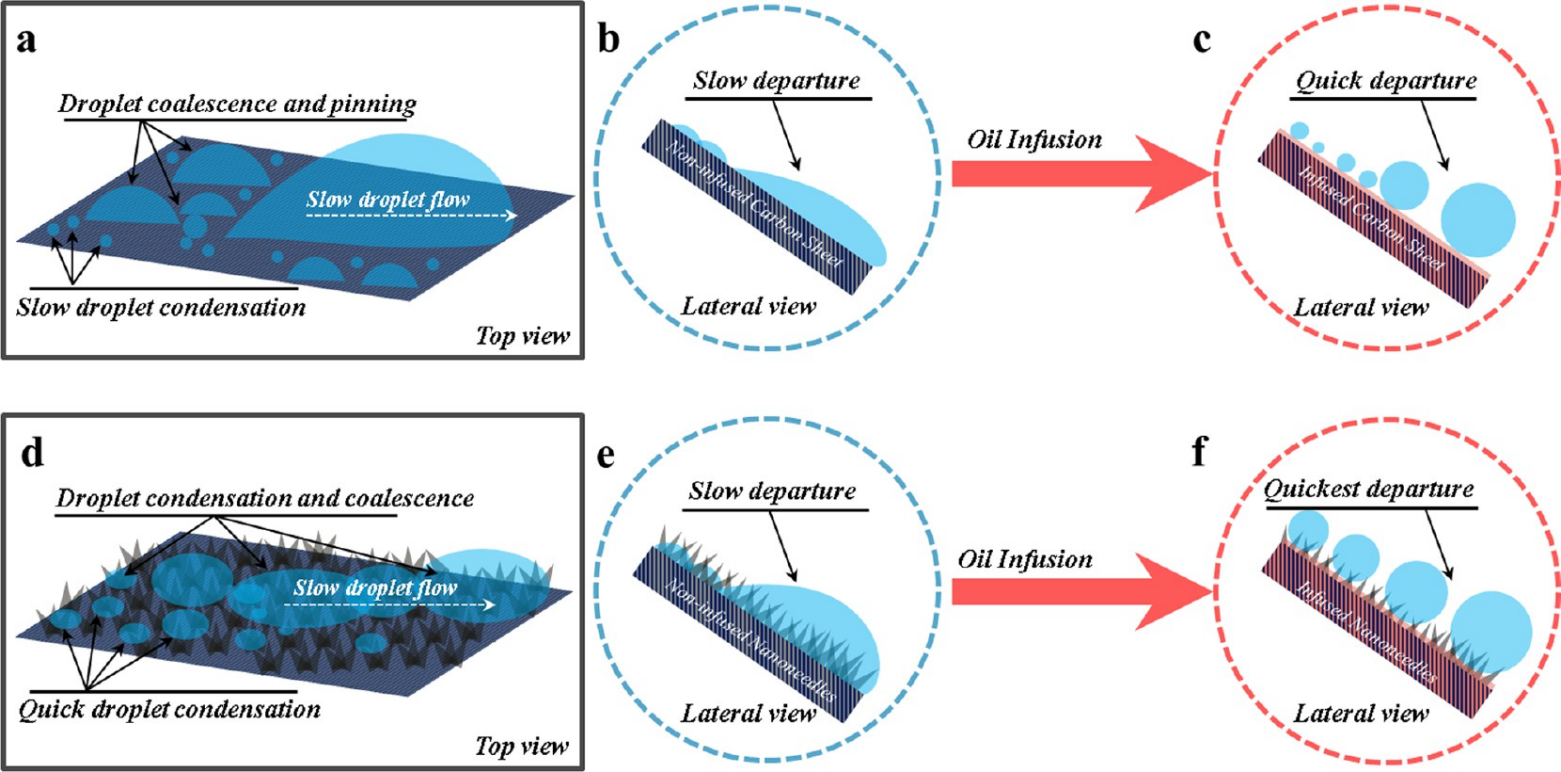
Mechanism of atmospheric water harvesting (AWH) and the condensed water movement. Schematic illustration depicting the AWH and condensed water movement mechanisms on carbon fiber and nanoneedle surfaces with and without liquid entrapment. Panels a–c show AWH on carbon fiber surfaces without liquid entrapped (WOLE) and with liquid entrapped (WLE), while panels d–f illustrate AWH on WOLE and WLE nanoneedle surfaces. Detailed mechanisms observed through optical microscopy are provided in Supporting Information S6.

Figure 14 illustrates the fog harvesting mechanism on the WOLE or WLE of carbon fiber and nanostructured surfaces. These visuals provide insights into how fog harvesting operates in this context. The structure and wetting characteristics of these surfaces are critical factors influencing their water harvesting capability. To gain a deeper understanding, we should also examine atmospheric water harvesting (AWH) on various surfaces, as shown in (Figure 9). Comparing these different scenarios is essential for assessing the effectiveness of fog harvesting techniques on different surface types.

To thoroughly understand this phenomenon, we investigated the fog collection process on two types of surfaces—those with and without oil infusion—using an optical microscope (Supporting Information S6). On surfaces without oil infusion, hydrophilic features facilitate the initial capture of fog droplets. These droplets gradually coalesce, forming larger droplets on the surface as fog continues to accumulate. This process involves the interplay of two forces governing droplet motion behaviors.^35^ The driving force is the wettability gradient, as described by eq 4.
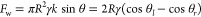
4variables *R* and *y* signify the droplet’s radius and surface tension of water; *θ*_l_ and *θ*_r_ denote the contact angles on the left and right sides of the droplet, following the variability in surface wetting characteristics. The hindrance is intertwined with the adhesive force (FA), and its connection to contact angle hysteresis (CAH). The overall horizontal force applied to the captured droplet can be expressed as *F* = *F*_w_ – *F*_A_ as per the *F*_w_ expression, with an increase in droplet volume, *F*_w_ systemically rises, ultimately exceeding *F*_A_, denoted by *F* > 0. As a result, the droplet is expected to proceed in the direction of the wettable gradient, accelerating surface renewal and increasing fog capture efficiency. The droplet on the hydrophilic zone spontaneously permeates the fiber or nanoneedle due to capillary forces outlined in eq 5.
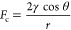
5which θ represents the water contact angle, and r denotes the average radius of carbon fiber/nanoneedles. After the droplet has entered the fibers/nanoneedles, the wettable gradient forces it is all directions of the surface. Given below eq 6:
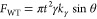
6

In which *t* is signified by the density of the nanoneedles and *k*_*y*_ corresponds to the wettable gradient in the density, both contributing to the accelerated droplet movement process. Eventually, the droplet sticks to the surface and enlarges by accumulating the droplets. Forming a large droplet.

The Laplace rule dictates that a small droplet’s high curvature radius creates greater pressure, driving flow from smaller to larger droplets, facilitating transmission. In the subsequent movement stage, when a captured droplet encounters a larger one on the surface, it is drawn downward by the internal Laplace pressure difference (LPD), easing further movement.^36^ However, on hydrophilic surfaces lacking efficient drainage mechanisms, hydrophilic nanoneedles may be overtaken by a water film, hindering fog collection circulation and efficiency. Conversely, on our liquid entrapped surfaces, captured water droplets on the hydrophobic surface are guided by the horizontal wettability gradient and move under the influence of an internal Laplace pressure difference, capillary force, and wettability gradient. This systematic cleaning of droplets from hydrophobic regions and the generation of new droplets enhance fog collection and surface regeneration rates, significantly boosting efficiency.

## Conclusion

In summary, we have successfully engineered advanced liquid-entrapped nanosurfaces optimized for highly effective atmospheric water harvesting (AWH). The achievement stems from a synergistic methodology that integrates carbon fiber paper (CFP) as a base substrate, hydrothermally synthesized nanoneedles (NNs) on CFP and the strategic inclusion of liquid entrapment (LE) of silicone oil within NNs. During the AWH process, CFP catches fog; however, it displayed pronounced water-pinning effects that would hinder the seamless movement of condensed water on the CFP, while NNs integrated with CPF exhibited improved droplet-spreading properties. The integration of NNs on CFP aimed at augmenting nucleation sites proved successful, resulting in a remarkable 50% rise in harvesting efficiency compared with surfaces lacking nanoneedles (i.e., CF alone). Upon entrapping silicone oil within CFP-bearing nanoneedles, the resulting surfaces (named liquid entrapped nanoneedles (LE-NNs) demonstrate exceptional efficiency in water harvesting compared to them without liquid entrapped counterparts (i.e., WOLE-NNs). Furthermore, upon oxidation, the nanoneedles become porous on the outer surface, upon silicon oil entrapment, the resultant surfaces (liquid entrapped oxidized nanoneedles (LE-ONNs)) reveal significant enhancement in fog harvesting, achieving an impressive water collection rate of 21.643 ± 0.538 L/m^2^/h, marking a 4-fold increase compared to CFP. These findings underscore the promising prospects for the practical implementation of our developed surfaces in fog harvesting and other liquid harvesting endeavors. By harnessing the unique properties of CFP, NNs, and ONNs, in conjunction with LE, our research paves the way for advancing water collection technologies and addressing critical challenges related to water scarcity and resource management.

## Supporting Information Available

S1: Video file of atmospheric water harvesting (AWH) performance on carbon fiber paper (CFP) and liquid-infused carbon fiber paper (LE-CFP) surfaces (MP4)
S2: Video file of atmospheric water harvesting (AWH) performance on nanoneedles (NNs) and liquid-infused nanoneedles (LE-NNs) surfaces (MP4)
S3: Video file of atmospheric water harvesting (AWH) performance on oxidized nanoneedles (ONNs) and liquid-infused oxidized nanoneedles (LE-ONNs) surfaces (MP4)
S4: Video file of the pinning and spreading behavior of a water droplet on carbon fiber paper (CFP) (MP4)
S5: Video file of the spreading behavior of a water droplet on nanoneedles (NNs) and oxidized nanoneedles (ONNs) surfaces (MP4)
S6: Video files illustrating the AWH mechanism on various surfaces with and without liquid entrapment (LE) (MP4)
